# Characterizing industry payments to ophthalmologists before and after onset of the COVID-19 pandemic

**DOI:** 10.21203/rs.3.rs-3329175/v1

**Published:** 2023-09-11

**Authors:** Hassaam S Choudhry, Hannaan S Choudhry, Carter Burton, Aman M Patel, Riya Patel, Ximin Li, Sean Berkowitz, Mona A Kaleem

**Affiliations:** Rutgers New Jersey Medical School; Rutgers New Jersey Medical School; Rutgers New Jersey Medical School; Rutgers New Jersey Medical School; Rutgers New Jersey Medical School; Johns Hopkins School of Public Health; Vanderbilt Eye Institute; Johns Hopkins Medicine Wilmer Eye Institute

## Abstract

**Purpose:**

The COVID-19 pandemic had profound effects on many different aspects of our healthcare system and the relationship between industry and physicians was no exception. The present database study evaluates industry payments to ophthalmologists in order to identify whether there are significant differences in industry payments to ophthalmologists before and after onset of the pandemic.

**Methods:**

The Centers for Medicare & Medicaid Services Open Payments Database was queried for all ophthalmologists who received industry payments between 2018 and 2021. Pre-Covid was defined as 2018–2019 while post-Covid was defined as 2020–2021. Payment date, value, type, company making payment, and state of recipient were recorded. The top ten companies and states in terms of payment value were included in analysis. Generalized Estimating Equations (GEE) modeling was used to assess significance.

**Results:**

There were 729,263 industry payments to 20,832 ophthalmologists totaling $817,892,867.54 included for analysis in this study. We found that there was a significant increase in the mean value of research payments and a significant decrease in the mean value of general payments after the onset of the pandemic (both p < 0.001). We also report significant changes in industry payments to ophthalmologists based on the company making the payment and the state in which the ophthalmologist practices.

**Conclusions:**

Our results suggest that significant differences exist in industry payment patterns to ophthalmologists following onset of the COVID-19 pandemic. Understanding underlying reasons for the observed differences may improve our understanding of the relationship between industry and clinical ophthalmology.

## A. Introduction

The short-term effects of the pandemic on both pharmaceutical industry corporations and healthcare workers are well documented [1, 2]. Given the extensive impact of the pandemic on both groups, of interest is characterizing industry payments to physicians, specifically ophthalmologists, before and after the COVID-19 pandemic. Inoue et al. found that, in the months of April to December of 2020, there was a significant decrease in industry payments to physicians relative to the months and years prior. Of note, the types of industry payments with the greatest decreases observed were predominantly in categories requiring physical contact, such as meals or travel [3].

The analysis of industry payments was only made possible a little over a decade ago when the Physician Payments Sunshine Act was passed into law in 2010 with the intention of increasing transparency of financial transactions between physicians and corporations [4, 5]. The national Open Payments Database, sponsored by the Centers for Medicare & Medicaid Services, exists as a direct product of this legislation, where all industry payments to physicians are recorded, including but not limited to the nature of payment, the corporation making the payment, and the value of payment [6].

As a result, the literature is extensive in ophthalmology regarding industry payment trends. Just recently in fact, Kalva et al. characterized industry payments to ophthalmologists for research from 2014 to 2020 [7]. Gender disparities and a lack of representation for female ophthalmologists receiving industry payments have been highlighted previously [8]. Despite this, it seems that no study has been published to date looking specifically at differences in industry payments to ophthalmologists and physicians following the onset of the COVID-19 pandemic. Hence, in this investigation we look to assess if significant discrepancies exist for industry payments made in 2020 and 2021 to ophthalmologists relative to payments made before the COVID-19 pandemic in 2018 and 2019.

## B. Methods

The Centers for Medicare & Medicaid Services Open Payments Database was queried for all ophthalmologists who received industry payments between 2018 and 2021. Pre-Covid was defined as 2018–2019 while post-Covid was defined as 2020–2021. Payment date, value, type, company making payment, and state of recipient were recorded. The top ten companies and states in terms of payment value were included in analysis. Generalized Estimating Equations (GEE) modeling was used to assess significance.

## C. Results

There were 729,263 industry payments to 20,832 ophthalmologists totaling $817,892,867.54 included for analysis in this study. Research-based payments composed of 9.07% of the number of payments and 71.40% of the total monetary amount of payments (Table 1). Among the non-research payments, food and beverages composed of the majority of payments (78.35% of total number of payments made), but represented only 2.53% of the monetary amount. After research payments, faculty or speakers was the classification with the greatest percentage of dollar amount paid (10.11%). Overall, the median payment was $22.21, and median research payment was $900.00.

Carl Zeiss was the company with the greatest mean value of payments made ($5,507.32) pre-COVID and Genentech had the greatest mean value post-COVID ($12,813.70) of all companies (Table 2). Genentech also had the largest median values for payments both pre-($92.63) and post-COVID ($2,700.85). Aside from AbbVie, Inc. and Johnson & Johnson, all top 10 companies had a statistically significant difference between their pre-COVID and post-COVID payments (p < 0.05). Alcon, Genentech, and Regeneron had significant increases in amount of payments, while Aerie Pharmaceuticals, Inc., Allergan, Inc., Bausch & Lomb, Carl Zeiss, and Novartis had significant decreases (p < 0.001). Genentech had the largest increase in mean difference (970.22), and Carl Zeiss had the largest decrease (17.92). Payments analyzed by classification also showed statistically significant differences between pre- and post-COVID amounts, with only “current or prospective ownership or investment interest” and grants not showing statistical differences between the two time periods (p > 0.05) (Table 3). Only education (8.48) and entertainment (210.06) showed significant increases in mean differences from pre-COVID to post-COVID payments (p < 0.001). Royalty or license payments had the largest decrease in mean difference (4,518.36) of all payment classifications.

When comparing payments made by the top 10 states, California had the greatest number of payments made both pre-(60,758) and post-COVID (48,041) (Table 4). Massachusetts had the largest median value pre-COVID ($32.29) and Texas had the largest median value post-COVID ($24.00). Furthermore, Massachusetts also had the greatest mean value of payments made pre-COVID ($1,365.36), while North Carolina had the greatest mean value post-COVID ($2,818.26). California (−5.25), Massachusetts (−11.11), and Pennsylvania (−8.33) showed significant decreases between pre- and post-COVID payment values (p < 0.05). Texas showed a statistically significant increase during that period (8.02, p < 0.001). All other top 10 states showed no statistically significant differences (p > 0.05).

## D. Discussion

The relationship between physicians and industry is a major source of public distrust in the American healthcare system. A 2016 survey of over 1,300 US adults showed that public disclosure of industry payments to physicians lowered patient trust, regardless of whether their physician had received any payments [9]. In a study from Hwong et al., people were more likely to perceive physicians receiving industry payments as dishonest, with physicians receiving higher payments being perceived as more dishonest [10]. Not only do industry payments to physicians seriously harm physician-patient relationships, but several studies also strongly suggest that industry payments can influence the care a patient receives [11, 12]. Recognizing trends in industry payments to physicians is vital for fully understanding the influence these payments can have both on public perception and the treatment physicians deliver. The COVID-19 pandemic is the greatest disruption the American healthcare system has seen in recent years. The pandemic has had a significant influence on the American healthcare system and healthcare expenditures [13]. Given this influence, this study aims to understand what differences, if any, exist for industry payments to ophthalmologists before and after onset of the COVID-19 pandemic. Several prior publications have used the Open Payments Database to address industry payments to ophthalmologists, and we look to specifically analyzed any changes in industry payments to ophthalmologists before and after the beginning of the COVID-19 pandemic made possible by the availability of 2021 data [4, 7, 14–16]. This study additionally serves as an update on general research payment trends to ophthalmologists.

Total industry payments to ophthalmologists from 2018 to 2021 amounted to over $800 million paid to 20,832 ophthalmologists (Table 1). The majority (71.4%) of the $800 million was for research-related payments, which includes direct compensation to physicians for research, funding for medical studies, and payments to study participants for study-related expenses. Research-related payments made up only 9% of the total number of payments and only 8% of all ophthalmologists received any research-related payment. This indicates that the majority of all money paid to ophthalmologists by industry was made in large payments to a relatively small number of ophthalmologists for research. This is consistent with another study from Tringale et al. showing that, compared to general payments, research payments to physicians regardless of specialty were of disproportionately larger value and were paid to a relatively small number of physicians [6]. Although the field of ophthalmology is reflective of payment trends seen across all physicians, the type and distribution of industry payments are field-dependent. For example, a study from Pakanati et al. excluded research payments when evaluating industry payments to hospitalists on the grounds that the number and value of payments were so small as to be insignificant [17]. The remainder (28.6%) of the $800 million paid to ophthalmologists by industry was in the general category (Table 1), which includes a broad range of subcategories such as consulting fees, food and beverages for work-related meetings, and gifts, among others. Within the general category, the two subcategories with the greatest value paid were consulting fees and faculty or speakers (8.59% and 10.11% of total paid, respectively), whereas the number of payments of these types made was relatively small (3.31% and 2.28% of all payments, respectively). Payments for food and beverage made up the majority of total payments made (78.35%), while being a small percentage of the total value paid (2.53%). Overall, this indicates that research payments are generally much greater than general payments, exemplified by comparing the median values of research and general payments ($900 and $21, respectively). This is consistent with other publications evaluating industry payments to physicians using the Open Payments Database [6, 18].

There was a significant increase in the mean payment to ophthalmologists after the beginning of the COVID-19 pandemic compared to before (Table 3). Notably, the *median* payment decreased slightly after the onset of the pandemic, suggesting that there was a relative increase in high-value payments. This increase in high-value payments is attributable to the notable increase in the mean research-related payment, which increased by 145% after the beginning of the pandemic. The number of research-related payments also increased. Conversely, there was a 25% decrease in the mean general payment after the pandemic and the number of general payments also decreased. Concordant with our findings, a study from Murayama et al. evaluating changes in general payments to rheumatologists pre- and post-pandemic found that the total value of general payments and the number of rheumatologists receiving general payments both decreased significantly after the onset of the pandemic [19]. Other publications reporting industry payments to plastic surgeons or infectious disease physicians before and after the onset of the pandemic show similar trends [20, 21]. Within the general category, the mean payment to ophthalmologists for consulting fees, education, faculty or speakers, food and beverage, gifts, honoraria, royalty or license, and travel and lodging all decreased significantly (p < 0.05). Social distancing and the cancellation of many in-person events explains drastic changes in payments made for food and beverage and travel and lodging, as many events moved to a virtual format. It is also likely that social distancing was the cause of changes in payments for consulting fees, education, and honoraria, as the shift to a virtual format decreased the cost of services rendered by ophthalmologists (and thus payments made to said physicians).

A study from Inoue et al. that evaluated industry payments to physicians in 2020 in the months before and after the onset of the pandemic had similar findings. The group found that there was a significant decrease in the mean value of industry payments to physicians for food and beverage, travel and lodging, consulting fees, faculty or speakers, and education and honoraria. Aside from food and beverage and travel and lodging, the group avoids speculating on a specific reason for changes in the value of payments [3]. The significant decrease in payments to ophthalmologists made for royalty or licensing fees, which are payments based on sales of products using a physician’s intellectual property, was likely due to a sharp decrease in medical device sales during the pandemic. Many hospitals redirected funding from nonessential equipment to personal protective equipment (PPE), ventilators, and other general hospital supplies [22, 23]. Conversely, the mean payment for entertainment increased significantly, although the number of entertainment-related payments dropped from over 600 to only 25 after the onset of the pandemic. This subcategory covers cost of attendance at recreational or sporting events, many of which were likely canceled or postponed after the onset of the pandemic. The increased mean payment for entertainment is likely explained by industry paying for more expensive events, which were less likely to be canceled in response to the pandemic.

Table 2 shows the top 10 companies making payments to ophthalmologists before and after the beginning of the pandemic. The *median* payment for all companies aside from Carl Zeiss, Genentech, and Regeneron decreased during the pandemic. The *mean* payment for all companies aside from AbbVie, Alcon, and Johnson and Johnson changed significantly during the pandemic, although there is no consistent direction of change amongst all companies. Based on these results, it seems that changes in payments made by the top 10 companies before and during the pandemic are company-dependent and followed no discernable trend.

Table 6 shows industry payments to ophthalmologists for the ten states with the highest total value of industry payments from 2018–2021. The high value of payments made to ophthalmologists in these ten states can likely be explained by each state’s population. Seven of these ten states are in the top-ten largest states by population and a greater population necessitates a greater number of physicians [24]. All ten states had a lower number of payments made to ophthalmologists during the pandemic compared to before. Interestingly, the only four states that had a significant change in mean industry payment to ophthalmologists (California, Massachusetts, Pennsylvania, and Texas) showed an increase in mean payment. As mentioned above regarding overall shifts in industry payments, it is likely that this is due to industry making a smaller number of larger-value payments, which would cause the mean payment value to increase.

Our study analysis is limited by the data collected in the Open Payments database. As such, conclusions about the COVID-19 pandemic definitively influencing industry payment patterns are limited without adjusting for potential confounders, and our analysis is restricted to simply describing the patterns observed. Additionally, inflation was not accounted for.

Understanding trends in industry payments to physicians is vital for understanding how the relationship between physicians and industry can influence public perception of the healthcare system and the way that physicians practice medicine. As one of the greatest disruptors of the American healthcare system in recent years, the onset of the COVID-19 pandemic may be associated with differences in industry payment patterns. This study evaluates changes in industry payments to ophthalmologists following onset of the COVID-19 pandemic. Future work in this field should aim to address changes in industry payments to ophthalmologists during the global recovery from the pandemic, as well as evaluate underlying reasons for the significant differences observed.

## Figures and Tables

**Table 1.** Classification and Amount of Payments Received by Ophthalmologists

Payment Classification	Total Payments in $, (% of total)	Number of Payments, (% of total)	Number of Ophthalmologists Receiving Payments, (% of total)	Median Payments in $, (IQR)
Overall	817,892,867.87 (100.00)	729,263 (100.00)	20,832 (100.00)	22.21 (15.25 – 111.98)
Research	583,995,449.33 (71.40)	66,117 (9.07)	1,678 (8.05)	900.00 (378.75 – 5,400.00)
General	233,897,418.54 (28.60)	663,146 (90.93)	20,670 (99.22)	20.69 (14.76 – 74.12)
Acquisitions	278,263.88 (0.03)	7 (0.00)	4 (0.02)	41,255.92 (24,232.16 – 56,894.68)
Charitable contribution	685,979.97 (0.08)	16 (0.00)	15 (0.07)	28,762 (2,085.61 – 49,974.25)
Consulting fee	70,289,098.46 (8.59)	24,119 (3.31)	2,379 (11.42)	1,500.00 (700.00 – 3,147.46)
Current or prospective ownership or investment interest	7,162,033.75 (0.88)	742 (0.10)	313 (1.50)	517.00 (288.91 – 1,895.00)
Debt forgiveness	132,806.24 (0.02)	52 (0.01)	34 (0.16)	392.16 (274.59 – 1,220.49)
Education	429,249.49 (0.05)	6,256 (0.86)	3,180 (15.26)	17.58 (8.22 – 39.95)
Entertainment	40,744.59 (< 0.01)	634 (0.09)	451 (2.16)	26.95 (16.70 – 67.48)
Faculty or speakers	82,650,813.94 (10.11)	16,639 (2.28)	2,101 (10.09)	2,250.00 (1,225.00 – 3,530.00)
Food and beverage	20,665,045.32 (2.53)	571,380 (78.35)	20,126 (96.61)	19.27 (14.21 – 30.58)
Gift	1,501,344.58 (0.18)	4,490 (0.62)	3,408 (16.36)	19.40 (19.40 – 45.00)
Grant	3,094,575.51 (0.38)	340 (0.05)	129 (0.62)	2,083.00 (1,377.01 – 5,000.00)
Honoraria	8,486,992.13 (1.04)	4,157 (0.57)	898 (4.31)	1,500.00 (735.00 – 2,550.00)
Long term medical supply or device loan	142,613.70 (0.02)	117 (0.02)	88 (0.42)	212.82 (97.46 – 1296.12)
Royalty or license	27,353,409.13 (3.34)	636 (0.09)	80 (0.38)	2,719.00 (340.00 – 18,033.50)
Travel and lodging	10,984,446.85 (1.34)	33,561 (4.60)	3,343 (16.05)	197.44 (54.13 – 400.00)

**Table 2.** Comparison of Payments Made by Top 10 Companies Types Pre- and Post-COVID

Company Name	Timeframe	Median Payments in $, (IQR)	Mean Payments in $, (SD)	Mean difference, (95% CI)	P-value
AbbVie, Inc.	Pre-COVID	68.86 (17.54 – 128.58)	2,781.10 (99,564.98)	−30.60 (−59.49, −1.72)	0.102
	Post-COVID	20.13 (13.81 – 98.71)	41,914.48 (380,262.40)		
Aerie Pharmaceuticals, Inc.	Pre-COVID	20.38 (14.68 – 104.11)	277.82 (2,547.76)	−7.67 (−9.97, −5.37)	**< 0.001**
	Post-COVID	19.33 (14.54 – 24.23)	208.67 (951.34)		
Alcon	Pre-COVID	58.22 (18.10 – 222.81)	580.25 (10,801.97)	8.42 (6.59, 10.24)	**< 0.001**
	Post-COVID	64.49 (18.96 – 344.4)	580.58 (9,673.24)		
Allergan, Inc.	Pre-COVID	21.82 (13.49 – 124.99)	815.06 (5,165.17)	−17.92 (−20.33, −15.52)	**< 0.001**
	Post-COVID	18.02 (12.91 – 39.95)	541.42 (4,891.82)		
Bausch & Lomb	Pre-COVID	21.39 (15.22 – 89.23)	319.24 (2,988.87)	−11.11 (−12.91, −9.31)	**< 0.001**
	Post-COVID	19.05 (14.78 – 23.51)	212.91 (2,453.20)		
Carl Zeiss	Pre-COVID	66.38 (26.84 – 152.21)	5,507.32 (283,875.23)	−41.99 (−48.65, −35.32)	**< 0.001**
	Post-COVID	19.40 (19.40 – 90.575)	552.33 (3,202.07)		
Genentech	Pre-COVID	92.63 (17.57 – 971.08)	3,837.73 (17,803.28)	970.22 (878.03, 1,062.40)	**< 0.001**
	Post-COVID	2,700.85 (28.86 – 12,587.625)	12,813.70 (31,807.33)		
Johnson & Johnson	Pre-COVID	35.00 (17.30 – 114.58)	694.57 (4,231.58)	5.27 (−0.32, 10.85)	0.057
	Post-COVID	29.24 (17.36 – 111.3175)	925.75 (4,766.43)		
Novartis	Pre-COVID	19.64 (14.32 – 70.99)	1,411.34 (17,882.6)	−5.38 (−7.70, −3.06)	**< 0.001**
	Post-COVID	18.48 (14.41 – 22.8425)	3,820.66 (35,685.38)		
Regeneron	Pre-COVID	64.25 (19.63 – 234.34)	2,034.81 (11,319.00)	19.41 (7.21, 31.60)	**0.001**
	Post-COVID	31.98 (19.19 – 1,020.00)	2,853.25 (15,252.62)		

**Table 3.** Comparison of Payments Made by Classification Type Pre- and Post-COVID

Payment Classification	Timeframe	Total Payments in $	Mean Payments in $, (SD)	Median Payments in $, (IQR)	Mean difference, (95% CI)	P-value
Overall	Overall	817892866.87	1128.01 (30705.81)	22.21 (15.25, 111.98)	−1.79 (−2.66, −0.92)	**< 0.001**
	Pre-COVID	368179471.27	902.72 (36886.46)	23.30 (15.29, 116.01)		
	Post-COVID	449713395.60	1417.66 (20138.96)	21.16 (15.22, 101.30)		
Research	Overall	583995449.33	8832.76 (48673.99)	900.00 (378.75, 5400.00)	−68.22 (−107.72, −28.72)	**< 0.001**
	Pre-COVID	216970012.05	7108.64 (35783.04)	1049.26 (471.28, 4400.00)		
	Post-COVID	367025437.28	10311.15 (57428.66)	752.00 (344.40, 6425.80)		
General	Overall	233897417.54	354.95 (28163.58)	20.69 (14.76, 74.12)	−6.28 (−6.97, −5.59)	**< 0.001**
	Pre-COVID	151209459.22	400.73 (36928.76)	21.82 (14.86, 95.87)		
	Post-COVID	82687958.32	293.61 (5361.57)	19.67 (14.65, 35.04)		
Acquisitions*	Overall	278263.88	39751.98 (22743.47)	41255.92 (24232.16, 56894.68)		
	Post-COVID	278263.88	39751.98 (22743.47)	41255.92 (24232.16, 56894.68)		
Charitable contribution	Overall	685979.97	42873.75 (48900.61)	28762.00 (2085.61, 49974.25)		
	Pre-COVID	685979.97	42873.75 (48900.61)	28762.00 (2085.61, 49974.25)		
Consulting fee	Overall	70289098.46	2968.16 (5496.53)	1500.00 (700.00, 3147.46)	−118.61 (−196.64, −40.59)	**0.003**
	Pre-COVID	36396972.35	3167.16 (5479.98)	1500.00 (700.00, 3660.00)		
	Post-COVID	33892126.11	2780.55 (5505.73)	1500.00 (750.00, 2800.00)		
Current or prospective ownership or investment interest	Overall	7162033.75	9961.10 (53977.12)	517.00 (288.91, 1895.00)	−158.99 (−390.09, 72.11)	0.179
	Pre-COVID	4577134.45	11273.73 (65355.89)	631.67 (316.00, 2000.00)		
	Post-COVID	2584899.30	8258.46 (33986.87)	481.52 (275.20, 1444.80)		
Debt forgiveness*	Overall	132806.24	2553.97 (10281.61)	392.16 (274.59, 1220.49)		
	Post-COVID	132806.24	2553.97 (10281.61)	392.16 (274.59, 1220.49)		
Education	Overall	429249.49	68.86 (476.18)	17.58 (8.22, 39.95)	8.48 (6.70, 10.27)	**< 0.001**
	Pre-COVID	209485.05	71.69 (598.61)	12.76 (7.89, 35.95)		
	Post-COVID	219764.44	66.35 (332.73)	39.95 (8.22, 39.95)		
Entertainment	Overall	40744.59	65.3 (151.36)	26.95 (16.70, 67.48)	210.06 (50.50, 369.61)	**< 0.001**
	Pre-COVID	30178.24	50.38 (92.66)	26.16 (16.46, 57.98)		
	Post-COVID	10566.35	422.65 (492.10)	235.00 (160.56, 313.33)		
Faculty or speakers	Overall	82650813.94	4997.63 (174766.49)	2250.00 (1225.00, 3530.00)	−210.74 (−324.99, −96.48)	**< 0.001**
	Pre-COVID	64763058.26	6818.60 (230517.88)	2500.00 (1500.00, 3800.00)		
	Post-COVID	17887755.68	2540.87 (7198.42)	1750.00 (1042.00, 2990.00)		
Food and beverage	Overall	20665045.32	36.31 (44.96)	19.27 (14.21, 30.58)	−4.28 (−4.48, −4.08)	**< 0.001**
	Pre-COVID	13167188.28	40.69 (47.97)	19.93 (14.29, 49.74)		
	Post-COVID	7497857.04	30.55 (39.91)	18.65 (14.13, 24.27)		
Gift	Overall	1501344.58	336.17 (6089.13)	19.40 (19.40, 45)	−312.54 (−398.18, −226.91)	**< 0.001**
	Pre-COVID	738697.53	2725.82 (13514.30)	389.00 (63.57, 1697.62)		
	Post-COVID	762647.05	181.80 (5227.13)	19.40 (19.40, 19.40)		
Grant	Overall	3094575.51	9101.69 (26922.12)	2083.00 (1377.01, 5000.00)	−891.42 (−1837.56, 54.72)	0.070
	Pre-COVID	1303114.83	7239.53 (24181.87)	2500.00 (1923.00, 6407.64)		
	Post-COVID	1791460.68	11196.63 (29642.36)	2083.00 (1000.00, 2625.00)		
Honoraria	Overall	8486992.13	2130.80 (5224.37)	1500.00 (735.00, 2550.00)	−426.46 (−537.82, −315.09)	**< 0.001**
	Pre-COVID	4430138.31	2758.49 (7902.82)	1500.00 (1000.00, 3500.00)		
	Post-COVID	4056853.82	1706.71 (1763.03)	1200.00 (735.00, 2200.00)		
Long term medical supply or device loan*	Overall	142613.70	1218.92 (2559.24)	212.82 (97.46, 1296.12)		
	Post-COVID	142613.70	1218.92 (2559.24)	212.82 (97.46, 1296.12)		
Royalty or license	Overall	27353409.13	51513.01 (150962.16)	2719.00 (340.00, 18033.5)	−4518.36 (−8469.02, −567.71)	**< 0.001**
	Pre-COVID	15608952.18	73976.08 (165917.05)	8550.00 (1792.63, 32364.42)		
	Post-COVID	11744456.95	36701.43 (138519.09)	1809.63 (200.00, 6858.81)		
Travel and lodging	Overall	10984446.85	337.15 (742.77)	197.44 (54.13, 400.00)	−25.50 (−38.46, −12.54)	**< 0.001**
	Pre-COVID	9298559.77	350.68 (789.46)	203.05 (57.72, 406.34)		
	Post-COVID	1685887.08	278.02 (484.39)	168.40 (44.00, 345.64)		

* P values were not recorded for payment classifications that did not have any value of payments either pre- or post- pandemic.

**Table 4.** Comparison of Payments by Top 10 States Pre- and Post-COVID

State of Recipient	Timeframe	Number of Payments	Median Payments in $, (IQR)	Mean Payments in $, (SD)	Mean difference, (95% CI)	P-value
CA	Pre-COVID	60,758	24.63 (16.74, 119.08)	1,136.51 (84,561.92)	−5.25 (−7.77, −2.73)	**< 0.001**
	Post-COVID	48,041	22.07 (15.76, 88.92)	1,534.87 (30,241.63)		
FL	Pre-COVID	41,028	25.40 (16.59, 125.00)	865.77 (11,214.89)	−3 (−6.38, 0.37)	0.083
	Post-COVID	33,530	22.72 (16.78, 122.76)	1,257.48 (10,189.69)		
MA	Pre-COVID	8,916	32.29 (18.14, 129.16)	1,365.36 (18,071.94)	−11.11 (−19.93, −2.29)	**0.019**
	Post-COVID	5,945	22.78 (15.83, 113.35)	2,683.94 (30,072.65)		
MD	Pre-COVID	8,630	21.94 (15.58, 103.86)	1,067.34 (9,383.60)	−0.76 (−6.32, 4.8)	0.790
	Post-COVID	7,034	20.14 (15.41, 45.00)	2,716.15 (21,553.90)		
NC	Pre-COVID	12,321	23.40 (14.99, 122.00)	997.43 (13,996.84)	4.96 (−1.26, 11.17)	0.113
	Post-COVID	8,384	21.26 (14.8575, 125.00)	2,818.26 (25,955.62)		
NY	Pre-COVID	33,686	21.42 (15.33, 92.50)	869.86 (52,587.76)	0.57 (−1.94, 3.08)	0.655
	Post-COVID	26,292	20.48 (15.49, 51.42)	781.15 (35,644.88)		
OH	Pre-COVID	14,075	22.93 (14.7, 122.57)	866.77 (6,333.87)	4.6 (−0.93, 10.13)	0.099
	Post-COVID	10,205	21 (15.21, 135.52)	1,442.73 (10,791.60)		
PA	Pre-COVID	19,234	21.44 (14.61, 112.50)	723.08 (6,464.86)	−8.33 (−11.92, −4.73)	**< 0.001**
	Post-COVID	13,980	19.40 (14.74, 47.73)	885.16 (7,702.77)		
TN	Pre-COVID	10,058	23.51 (15.57, 121.65)	1,235.57 (14,527.56)	5 (−5.47, 15.48)	0.346
	Post-COVID	8,152	22.01 (15.90, 185.94)	1,825.59 (18,393.26)		
TX	Pre-COVID	34,413	25.94 (16.71, 125.00)	1,163.58 (10,213.38)	8.02 (4.25, 11.79)	**< 0.001**
	Post-COVID	28,178	24.00 (17.14, 282.90)	2,226.20 (18,936.89)		
